# Bolus *versus* continuous insulin infusion in immediate postoperative blood glucose control in liver transplantation: pragmatic clinical trial

**DOI:** 10.31744/einstein_journal/2022AO6959

**Published:** 2022-05-24

**Authors:** Luciana Vládia Carvalhêdo Fragoso, Márcio Flávio Moura de Araújo, Lidianne Fernandes da Silva Lobo, Dirk Schreen, Maria Lúcia Zanetti, Marta Maria Coelho Damasceno

**Affiliations:** 1 Universidade Federal do Ceará Hospital Universitário Walter Cantídio Fortaleza CE Brazil Hospital Universitário Walter Cantídio, Universidade Federal do Ceará, Fortaleza, CE, Brazil.; 2 Fundação Oswaldo Cruz Eusébio CE Brazil Fundação Oswaldo Cruz, Eusébio, CE, Brazil.; 3 Universidade de São Paulo Escola de Enfermagem de Ribeirão Preto Ribeirão Preto SP Brazil Escola de Enfermagem de Ribeirão Preto, Universidade de São Paulo, Ribeirão Preto, SP, Brazil.; 4 Universidade Federal do Ceará Fortaleza CE Brazil Universidade Federal do Ceará, Fortaleza, CE, Brazil.

**Keywords:** Liver transplantation, Postoperative period, Insulin infusion systems, Glycemic control

## Abstract

**Objective:**

To analyze the effectiveness and safety of two insulin therapy techniques (continuous and intermittent infusion) in the blood glucose control of people who have undergone liver transplantation, in the immediate postoperative period.

**Methods:**

The study was a prospective, open, pragmatic clinical trial with 42 participants, divided into two groups of 21 patients each, in the immediate postoperative period following liver transplantation. Participants in the Experimental Group and Control Group received continuous infusion and bolus insulin, respectively, starting at capillary blood glucose ≥150mg/dL.

**Results:**

There were no statistically significant differences in the blood glucose reduction time to reach the target range between the Experimental Group and Control Group in the transplanted patients (p=0.919). No statistically significant differences regarding the presence of low blood glucose (p=0.500) and in the initial blood glucose value (p=0.345) were found. The study identified the final blood glucose value in postoperative intensive care unit lower and statistically significant in the continuous infusion pump group in relation to the Bolus Group (p<0.001). Additionally, the variation of blood glucose reduction was higher and statistically significant in the continuous method group (p<0.05).

**Conclusion:**

The continuous infusion method was more effective in the blood glucose control of patients in the postoperative period following liver transplantation.

**Brazilian Registry of Clinical Trials:**

RBR-9Y5tbp

## INTRODUCTION

The relevant literature presents wide-ranging discourse about the causes, harmful effects, glucose rate monitoring, established target ranges, control of high blood glucose, and drug therapy in critically ill patients in intensive care units (ICU). In this scenario, those who received heart transplant, kidney transplant and double transplant were included, as well as those who underwent cardiac surgery or who suffered brain damage or trauma.^(1–9)^ However, studies that specifically address the causes and adverse effects of high blood glucose, as well as the various aspects of blood glucose control in patients in the immediate postoperative period after liver transplantation are still scarce.^(3,10–13)^

Regarding liver transplantation, the desired blood glucose control can be rigid or malleable. The first one comprises the target range of 80-110mg/dL or lower than 150mg/dL. The second allows a rate of 140-180mg/dL.^(10–14)^

Blood glucose control in the postoperative phase can be performed by the administration of insulin in continuous infusion or bolus, also known as the sliding scale method.^(3,10–13)^ In both, the route is necessarily intravenous. Use of subcutaneous insulin to normalize the blood glucose rate in postoperative patients still in the ICU is rarely mentioned.^(15)^

Insulin administration requires the use of a continuous infusion pump (CIP) as well as a carefully prepared insulin solution, requiring correct scheduling of ejection velocity according to the blood glucose rate. In the bolus method, insulin is administered in intermittent doses that also vary according to the blood glucose rate.^(16)^

However, not all institutions have protocols that establish methods of insulin administration for blood glucose control of liver transplant patients during ICU stay. Thus, in clinical practice, both the bolus method and the continuous infusion method have been used at one time or another.

The benefits of continuous insulin administration in liver transplant patients were recorded in previous clinical investigations.^(3,11,13,17)^ Among critical patients in general, it was found that insulin administration by CIP reduced morbidity and mortality.^(18)^ However, in the Normoglycemia in Intensive Care Evaluation and Survival Using Glucose Algorithm Regulation study in 2009, conducted on people suffering from brain trauma, it was evidenced that the group which received insulin with an intensive method had a higher incidence of low blood glucose and percentage of mortality than the group that received intermittent doses of insulin.^(19)^ The controversies between continuous insulin and bolus insulin for blood glucose control, in critical care units, come from studies involving people in different health situations. Hence, the effectiveness of both methods is still little known, especially in the case of liver transplants.

## OBJECTIVE

To analyze the effectiveness and safety of two insulin therapy techniques (continuous and intermittent infusion) in the blood glucose control of people who have undergone liver transplantation, in the immediate postoperative period.

## METHODS

### Design and location of the study

This pragmatic, open-label, randomized clinical trial was developed at a federal public hospital, responsible for health care in outpatient, surgical, medical and intensive care settings, in the city of Fortaleza, CE, Brazil. It should also be noted that this hospital is among the three largest providers of liver transplant services in Brazil. This research has received financial support from the National Council for Scientific and Technological Development (CNPq - *Conselho Nacional de Desenvolvimento Científico e Tecnológico*) of Brazil according to process # 477747/2012-4.

### Participants

#### Eligibility criteria

The study stipulated the following criteria for participant inclusion: patient submitted to liver transplantation, regardless of sex; age ≥18 years; and capillary blood glucose ≥150mg/dL. This value was based on the recommendations of Jacobi et al.^(20)^ who states this target range as the ideal for initiating insulin therapy and reducing mortality in patients undergoing intensive and/or surgical care. The following were excluded from the trial: patients with severe graft dysfunction and/or death within the first 48 hours; patients with double transplantation (liver and kidney); patients with acute retransplantation in 48 hours; in addition to those using norepinephrine above 1*μ*g/kg/min.^(20)^

### Interventions

After the liver transplantation was completed, the patient was transported to the postoperative ICU for the following clinical care: monitoring of vital signs and/or bleeding, rigorous water balance, collection of laboratory tests, oxygen therapy (via mechanical ventilation or support device of specific oxygenation), and blood glucose control.

During the first 24 hours, in order to guarantee blood glucose control within the target range of <150mg/dL, a blood glucose meter and capillary puncture were used every hour by the nursing staff. Thus, after blood glucose measurement, those included in the inclusion criteria were submitted to one of two interventions, namely: bolus insulin administration or CIP.

### Control Group Intervention (bolus- intermittent intravenous insulin administration)

The Control Group (bolus-intermittent intravenous insulin administration), based on blood glucose value (≥150mg/dL), used the following sliding insulin regimen: blood glucose at 150-200mg/dL (4IU bolus insulin); 201-250mg/dL (8IU bolus insulin); 251-300mg/dL (12IU bolus insulin); and >300mg/dL (15IU bolus insulin). The injectable solution of regular human insulin available in the service was Novolin R vial, with 100IU/mL manufactured by Novo Nordisk Farm do Brasil LTDA^®^.

### Experimental Group Intervention (intravenous infusion of insulin by CIP)

The Experimental Group, based on blood glucose value (≥150mg/dL), used the continuous insulin therapy regimen: dilution of 100IU of regular insulin in 100mL of 0.9% saline solution. Subsequently, the patient’s blood glucose value was divided by 100, and its result rounded to decimal values at 0.50. This value determined the initial flow of the infusion pump. When the blood glucose target between 100 and 150mg/dL was reached and maintained for three consecutive hours, the team began weaning the insulin flow rate at 1mL/h, in order to prevent cases of low blood glucose. However, blood glucose control (mediated by capillary blood glucose) persisted, with the possibility of resuming insulin therapy, if necessary.

The injectable solution of regular human insulin available in the service is Novolin R vial, with 100IU/mL, manufactured by Novo Nordisk Farm do Brasil LTDA^®^.

### Measurements

Identification variables (sex and age) and clinical variables were observed. These were divided into diagnosis (hepatitis B, C and D, autoimmune hepatitis, alcoholic cirrhosis, cryptogenic cirrhosis etc.); comorbidities (ascites, encephalopathies, esophageal varices, diabetes, hypertension, alcohol abuse, smoking, obesity etc.); drugs in use (antimicrobials, diuretics, gastric protector, cardioprotective drugs, corticoids, antiretroviral etc.), cold ischemia time (hours); warm ischemia time (minutes); FiO_2_ - inspired fraction of oxygen (percentage); mechanical ventilation (controlled ventilation – volume or pressure, pressure-assisted ventilation, continuous positive air pressure), and laboratory data (C-reactive protein - CRP, lactate dehydrogenase - LDH, and lactic acid).

Additionally, the Model for End-Stage Liver Disease (MELD) (<10 points, 10-19 points, 20-29 points, 30-39 points, and ≥40 points) and the Simplified Acute Physiology Score 3 (SAPS 3) (point scores) were applied.

As outcomes, the following variables were measured: blood glucose reduction (characterized in mg/dL), blood glucose reduction coefficient (characterized by ratio between blood glucose per hour), low blood glucose (characterized by blood glucose <70mg/dL), days in ICU, use of vasoactive drugs, mortality, infection, rejection, and hemodialysis.

### Population and sample

The study population consisted of patients in the immediate postoperative period following liver transplantation, of both sexes, aged 18 years old and over, and admitted to the postoperative ICU. The period up to 24 hours after the surgery was considered “immediate”.

**Figure 1:**
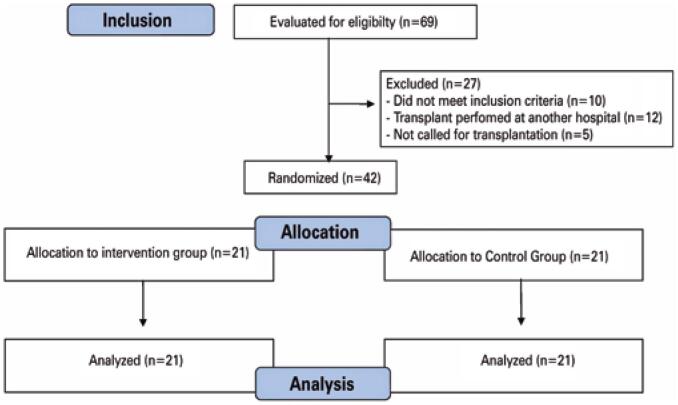
Flowchart of study participants

Based on a 95% confidence interval, 80% power and variance of 1.73mg/dL of blood glucose between the bolus and CIP techniques, this study considered that out of the 21 pairs per group, it would be possible to establish a mean difference between groups in the capillary blood glucose outcome. The final sample consisted of 42 liver transplant patients admitted to the ICU from September 2015 to February 2016 (Figure 1).

### Recruitment and randomization of participants

Eligible participants were initially approached at the liver transplant outpatient clinic. Subjects who agreed to participate in the study signed the Informed Consent Form and were given a copy of said form.

Through a design by the researcher, the participants were randomly divided into two groups: the Experimental Group (CIP) and the Control Group (Bolus). The homogeneity of the groups was assumed based on the absence of statistically significant differences between the age groups (p=0.252) and the severity of liver disease according to the MELD (p=0.564) and SAPS (0.970) criteria.

### Data collection

A blood glucose monitor that was adopted in the measurements was designated exclusively for this function; the device underwent monthly preventive maintenance at the study site.

The researchers monitored the groups data through daily visits to the postoperative ICU. In turn, the on-duty medical and nursing teams performed the interventions of the aforementioned groups, according to the established protocols. Therefore, nursing professionals were trained (with a total duration of 16 hours) and meetings were held with the medical team to ensure uniformity in these steps.

The following topics were addressed: inclusion and exclusion criteria for initiating the protocol; blood glucose measurement; preparation, administration and monitoring of insulin therapy (signs of low blood glucose, dose adjustment, rotation of the capillary puncture and peripheral perfusion).

### Prevention of adverse events

Strategies to minimize the risk of low blood glucose were also developed, according to the blood glucose outcome. In the cases where the blood glucose reduction was <30%, the infusion was maintained. In situations of reduction >30%, the flow rate was reduced by half.

### Data analysis

Data were expressed as mean, standard deviation, median, 25^th^ and 75^th^ percentile for scalar variables, and in frequency and percentage for categorical variables. After data normality analysis, by means of the Shapiro-Wilk test, we used the Mann-Whitney test for comparisons between the groups. We adopted a significance level of 5%. Statistical analyses were performed using SPSS, version 22.0 (USA) and R 3.3.1 software. We also constructed a regression line by the method of least squares, with the outcome variable being the blood glucose level and time predictor (hours).

### Protection of participants

The study was approved by the Research Ethics Committee of the *Universidade Federal do Ceará* (UFC), # 1.063.210, CAAE: 41941115.1.0000.5054. *Hospital Universitário Walter Cantídio* # 1.107.776, CAAE: 41941115.1.3001.5045, Brazilian Registry of Clinical Trials (Rebec), under # RBR-9Y5tbp.

## RESULTS

### Characterization of participants

Sixty-two percent were men and alcoholics, ranging from 54 (±14) to 58 (±9) years old; 16.6% were diabetics who used insulin (21.4%); and all had cirrhosis. There was a predominance of alcoholic hepatitis (33.3%) and type 2 diabetes (21.4%). Regarding liver disorders, the following types of hepatitis were predominant: B (12%), C (31%), D (4.7%), autoimmune (7.1%), cryptogenic (16.6%) and sclerosing cholangitis (4.7%). The main complications related to liver disease were encephalopathy (40.4%), ascites (31%), esophageal varices (14.2%), portal hypertension (7.1%), and hepatocellular carcinoma (38.1%) (Table 1).

**Table 1:** Baseline characteristics of participants in relation to clinical characteristics

Clinical variables	CIP Group n (%)	Bolus Group n (%)	p value
Sex			
Male	13 (62.0)	13 (62.0)	1.000
Age (years) (mean±SD)	58±9	54±14	0.786
*Diabetes mellitus*	9 (42.8)	8 (38.0)	1.000
Hypertension	6 (28.5)	8 (38)	0.743
Obesity	4 (19)	1 (4)	0.343
Smoking	9 (42.8)	4 (19)	0.182
Alcohol abuse	13 (62)	13 (62)	1.000
Hepatic morbidities			
Alcoholic hepatitis	7 (33.3)	7 (33.3)	1.000
Hepatitis C	7 (33.3)	6 (28.5)	1.000
Hepatocellular carcinoma	9 (42.8)	7 (33.3)	1.000
Encephalopathy	9 (42.8)	8 (38)	1.000
Ascites	6 (28.5)	7 (33.3)	1.000
Hepatic cirrhosis	21 (100)	21 (100)	

SD: standard deviation; CIP: continuous infusion pump.

The study did not identify statistically significant differences between the groups for age (p=0.252), cold ischemia time (p=0.435) and FiO_2_ (p=0.319). The Experimental Group and the Control Group were also homogeneous regarding the severity level of liver disease, according to the MELD criteria (p=0.564) and SAPS criteria (0.970).

### Blood glucose reduction

In general, the participants presented reduced blood glucose and elevated insulin. The blood glucose reduction in the Experimental Group up to the target range was higher (41%) and lasted until the final measurement (25%). In the Control Group, the reduction up to the target range was lower (34.8%), and there was a blood glucose increase (9.3%) until the final measurement (p<0.001). Before initiating the intervention, the concentration of insulin administered initially was higher in the Control Group (p<0.001).

Only four patients (9.5%) had episodes of low blood glucose. Of these, three (75%) were from the Experimental Group. The study did not find statistically significant differences as for the presence of low blood glucose (p=0.500) and initial blood glucose value (p=0.345) in the groups (Table 2).

**Table 2:** Blood glucose control of participants in general and between groups

	General					CIP Group					Bolus Group				
Variables	Mean	Median	SD(±)	P_25_	P_75_	MD	MED	SP(±)	P_25_	P_75_	MD	MED	SD(±)	P_25_	P_75_
IBGV (mg/dL)	218.3	204.0	65	176.0	248.0	225.9	206.0	72.25	185.0	248.0	210.8	190.0	57.5	174.0	219.0
BGVRT (mg/dL)	135.7	138.0	11.3	130.0	144.0	134.0	135.0	12.5	129.0	144.0	137.5	139	9.9	130.0	143.0
FBGV (mg/dL)	126.0	119.5	42.1	99.0	138.0	101.7	99.0	30.9	89.0	118.0	150.3	136.0	38.0	127.0	176.0
IIV (unit-UI and mL/h)	4.7	4.0	3.8	2.0	4.0	2.3	2.0	0.5	2.0	2.5	7.1	4.0	4.2	4.0	8.0
IVRT (mL/h)	8.7	6.5	6.9	4.0	10.0	8.7	6.5	6.9	4.0	10.0	-	-	-	-	-
FIV (UI and mL/h)	6.5	4.0	5.2	3.0	8.0	6.6	4.0	6.0	3.0	11.0	6.0	6.0	2.1	4.0	8.0
LBGV (mg/dL)	62	64	80	57	67	65	65	30	63	68	50	50	-	50	50

IBGV: Initial blood glucose value; BGVRT: blood glucose value upon reaching target; FBGV: final blood glucose value; IIV: initial insulin value; IVRT: insulin value upon reaching target; FIV: final insulin value; LBGV: low blood glucose value; SD: standard deviation; P_25_: percentile _25_; P_75_: percentile _75_; CIP: continuous infusion pump.

The study found that the final value (mean, median, and standard deviation) of blood glucose in the postoperative phase was lower and statistically significant in the Experimental Group (102/99±31mg/dL) in relation to the Control Group (150/136±36mg/dL) (p<0.001). Moreover, the variation of blood glucose reduction was higher and statistically significant in the participants from the Experimental Group (p<0.05) (Table 3).

**Table 3:** Blood glucose reduction rate per hour, according to the groups

Variables	CIP Group		Bolus Group		p value
	Median	Standard deviation	Median	Standard deviation	
Blood glucose reduction rate (mg/dL)	14.2	8.62	12.9	11.9	0.285
Variation (%)	0.45	0.47	0.05	0.26	<0.05

Variation: initial value minus final value when desired target range was reached.

CIP: continuous infusion pump.

The study did not find statistically significant differences in the time of blood glucose reduction until the target range between the patients in the Experimental Group and Control Group in the liver transplantation postoperative period (p=0.919).

### Blood glucose control

Among people with diabetes, no statistically significant differences were found in the rate of blood glucose reduction (p=0.547), percentage of blood glucose variation (p=0.918) and days of hospital stay (p=0.319). Among the hypertensive patients, the same behavior was observed, with no statistical relevance, in the aforementioned variables (p=0.626, p=0.228, and p=0.488).

Up to the sixth hour of blood glucose monitoring, the following statistically significant trends were found: from the second hour, there was a difference between the groups (p=0.030); in the CIP Group, blood glucose increased positively (10.7mg/dL per hour); the Bolus Group had a trend of lower blood glucose growth (p=1.4mg/dL per hour).

No significant statistical associations were found between graft rejection (p=0.311), hemodialysis (p=0.549), vasoactive drug use (p=0.726), and interventions instituted in this clinical trial. Only one patient presented acute rejection (Bolus Group) and rehospitalization within 30 days. Up to the end of data collection, three deaths occurred in the Control Group: two due to severe graft dysfunction and one due to bloodstream infection. There was no death in CIP Group.

The study showed no statistically significant differences in the monitoring of infection markers such as LDH (p=0.968), lactic acid (p=0.133) and CRP (p=0.669) between the groups, nor did it show any statistically significant association between the insulin therapy techniques used and the diagnosis of nosocomial infection (p=0.726), and length of hospital stay (p=0.322).

## DISCUSSION

Based on the findings of this study, it cannot be inferred that one method is more effective than the other in the time to reach the target range.

The authors who reported a faster time to reach the target range in using CIP focused on participants with heart disease and clinical patients.^(13,15,21)^ The patients’ condition in this study (severe liver disease) may bear some relation to this finding, since the liver is known to be vital for insulin resistance and blood glucose control.

An extensive review of the literature on blood glucose control in intensive care and the use of insulin therapy protocols concluded that these resources significantly improve patients’ overall health, because they reduce the time to normoglycemia and help maintain it. The authors also found that the CIP was safer and more efficient than the (conventional) sliding scale method.^(22)^

In this research, the continuous infusion method showed greater variation (rate of blood glucose reduction - mg/dL per hour). Additionally, the final blood glucose value of the continuous infusion method was lower than the intermittent one. Therefore, based on these findings, the continuous infusion method, in the studied group, is believed to be more effective than the intermittent method. On the other hand, a study with a focus on blood glucose control in intra and postoperative phases, conducted with people who have undergone liver transplantation, revealed that sometimes the bolus was necessary to control high blood glucose (even in those using CIP).^(11)^

Regardless of the strategy, there is consensus among researchers that blood glucose control should be rigorous in people who have undergone liver transplantation in the postoperative phase.^(3,15,23)^

Regarding the outcome related to patient safety, characterized by low blood glucose, no differences were found between the insulin therapy methods used in this study. Previous studies consulted support this controversy in the literature.^(13,18,24–29)^

Despite the negative, non-significant results, it is important to discuss this topic - low blood glucose in the postoperative period - given the clinical relevance and the inductive error of the p value.^(30)^

One of the factors that may have influenced the cases of low blood glucose was the establishment of a control with a more rigid target range (<150mg/dL in this study).^(28,31)^ Another explanation would be the occurrence of the phenomenon of insulin adsorption in those using CIP. In other words, the insulin - by spending more time in contact with venous infusion lines - would have its action delayed and, consequently, fewer cases of low blood glucose.

A limitation of this research was the use of portable blood glucose monitors for measurement (less accuracy in relation to other measurements, such as plasma glucose). Despite arterial blood being more reliable than peripheral blood, arterial devices may increase the risk of infections and leave the patients skin weaker.^(27,32)^

A secondary concern of the researchers in this study was to observe the trend of blood glucose after reaching the target range, according to the insulin therapy methods. In this case, the intermittent technique showed stable behavior in the six hours after the target range was reached.

However, this finding should be analyzed with caution, since - after blood glucose remained within the target range (100 to 150mg/dL) for three consecutive measurements - weaning started at 1mL/h of continuous insulin flow. Moreover, when blood glucose reached values lower than 100mg/dL, the CIP was switched off to prevent low blood glucose. Thus, the study concluded that by around the fifth hour, blood glucose exceeded the maximum target range (150mg/dL), and the CIP was resumed.

Clinicians agree that it is imperative for appropriate blood glucose control to be initiated after stabilization of the condition, in order to avoid large oscillations in blood glucose, which is as harmful or even more so than high blood glucose itself.^(31)^ Regarding the present study, it is worth noting that although there was no statistical significance, no patient died in the Experimental Group and, in relation to the severity score (SAPS 3 score), the groups were similar.

One limitation of this investigation was not having observed the performance of the insulin therapy methods according to the medical and nursing staff on duty. Even with meetings and specific training, variations associated with the management of each team may have occurred. However, some authors argue that the monitoring of blood glucose by nurses is as effective and safe as the intermittent method (bolus).^(22)^

Thus, the specific training of these healthcare professionals is important in services that lack technical inputs relating to the blood glucose control of patients who have undergone liver transplantation in the immediate postoperative period.

Studies concerned with the comparison and/or evaluation of blood glucose control methods in critical patients can cooperate to promote a culture of patient safety in ICU. It is important for critical care physicians and nurses to be familiar with the best blood glucose control technique in the postoperative phase following liver transplantation, in order to minimize complications such as high blood glucose, low blood glucose, infection, and loss of transplant, or even an increase in hospitalization and correlated medical procedures, since they are responsible for the preparation, administration and monitoring of the inputs involved.

Future studies with a larger sample and more rigorous designs that culminate in more robust evidence are suggested to improve the clinical practice of people undergoing liver transplantation.

## CONCLUSION

Administration of insulin by continuous infusion presented better results regarding the variables “blood glucose variation” and “final blood glucose value.” Therefore, the results of this study show that it has been the most effective method for the administration of bolus insulin for blood glucose control in the immediate postoperative period following liver transplantation.
